# Understanding self-harm and suicidal behaviours in South Asian communities in the UK: systematic review and meta-synthesis

**DOI:** 10.1192/bjo.2023.63

**Published:** 2023-05-15

**Authors:** Büşra Özen-Dursun, Safa Kemal Kaptan, Sally Giles, Nusrat Husain, Maria Panagioti

**Affiliations:** Division of Psychology and Mental Health, School of Health Sciences, Faculty of Biology, Medicine and Health, University of Manchester, Manchester Academic Health Science Centre, UK; Global Mental Health and Cultural Psychiatry Research Group, University of Manchester, UK; and Department of Psychology, Boğaziçi University, Türkiye; Division of Population Health, Health Services Research and Primary Care, School of Health Sciences, National Institute for Health and Care Research Greater Manchester Patient Safety Translational Research Centre, Faculty of Biology, Medicine and Health, University of Manchester, UK; Division of Psychology and Mental Health, School of Health Sciences, Faculty of Biology, Medicine and Health, University of Manchester, Manchester Academic Health Science Centre, UK; and Mersey Care NHS Foundation Trust, Prescot, UK; Division of Population Health, Health Services Research and Primary Care, School of Health Sciences, National Institute for Health and Care Research Greater Manchester Patient Safety Translational Research Centre, Faculty of Biology, Medicine and Health, University of Manchester, UK; and Division of Population Health, Health Services Research and Primary Care, School of Health Sciences, National Institute for Health and Care Research School for Primary Care Research, Faculty of Biology, Medicine and Health, University of Manchester, UK

**Keywords:** Self-harm, suicidal behaviour, South Asian, UK, meta-ethnography

## Abstract

**Background:**

Previous findings have indicated that self-harm and suicide are associated with different rates, and different risk and protective factors in South Asian people compared with White people in the UK. Substantial qualitative research has explored experiences of self-harm and suicide in South Asian people.

**Aims:**

The study aims to review the existing qualitative evidence on self-harm and suicidal behaviours in South Asian communities in the UK.

**Method:**

Systematic searches were conducted on Medline, EMBASE, PsycINFO, CINAHL, Open Dissertations and the British Library Ethos databases. We selected qualitative studies from both journals and grey literature that included South Asian participants who were resident in the UK and presented perceptions or experiences of self-harm and/or suicidal behaviour. Analysis was undertaken based on the meta-ethnographic approach.

**Results:**

Fifteen studies were included in the analysis. Experience of self-harm was discussed based on three aspects: behind self-harm, functions of self-harm and recovery from self-harm. ‘Behind self-harm’ refers to factors associated with self-harm and suicide. ‘Functions of self-harm’ captures the meaning attributed to self-harm and suicide. ‘Recovery from self-harm’ encapsulates personal and professional help, and practical suggestions for the improvement of mental health services.

**Conclusions:**

Although some similarities with the majority White population were present, there were also crucial differences that need consideration when shaping health policies, improving access to health services and developing culturally sensitive psychosocial interventions for self-harm and suicide specific to South Asian communities in the UK**.**

## Background

Suicide is a critical public health concern and one of the leading causes of death in the UK and across the world.^1,2^ Epidemiological studies have reported that more than 700 000 people lose their lives by suicide each year globally,^1^ with 5583 suicides recorded in 2021 in England and Wales.^2^ Moreover, the risk of suicide is higher in people with history of self-harm compared with those without such history.^3^ Therefore, the associations between self-harm and suicide have been widely studied.^4^

Clinical record studies have shown that suicide rates and risk factors for suicidal behaviours vary considerably in ethnic minority groups in England and Wales.^5–7^ Similarly, self-harm rates differ across the ethnic groups in the UK.^8^ The self-harm rate was higher among South Asian women than White women aged 16–24 years, but was lower in South Asian men than in White men in all age groups.^9^ Further, older South Asian people were found to be a high-risk group for suicide^7^ and depression.^10^ An observational cohort study indicated that the rate of hospital presentation of self-harm was lower in ethnic minority children and adolescents compared with their White counterparts.^11^ However, rates of self-harm increased more in the ethnic groups than in the White group across the 16-year study period.^11^ Further, ethnic minority groups were less likely to receive a specialist psychosocial assessment by psychiatry liaison staff.^11^ This evidence is reflected in the National Suicide Prevention Strategy for England, which highlights high-risk groups, such as Black, Asian and other minority ethnic groups and people with a history of self-harm. More research is needed to understand the mechanism of self-harm, and more actions to deliver tailored suicide prevention approaches for high-risk group are encouraged.^12^

Previous literature reviews have evaluated the clinical characteristics of, and risk factors for self-harm among ethnic communities in the UK.^8,13,14^ The review of self-harm in British South Asian women has illustrated that cultural conflict and marital and interpersonal problems could have major influences on self-harm.^14^ However, these reviews are outdated, focusing mostly on quantitative studies and often including heterogeneous ethnic minority samples, thereby precluding inferences focused on South Asian people.^8,13^ Therefore, synthesis of in-depth and methodologically strong evidence examining the experiences of self-harm among South Asian people is lacking. This study is the first systematic review of qualitative studies to employ a meta-ethnographic synthesis to examine how self-harm and suicide are viewed or experienced by South Asian people in the UK, including perceptions about risk factors, the recovery process and mental health service responses.

## Method

This systematic review was guided by and presented according to the Preferred Reporting Items for Systematic Reviews and Meta-Analysis (PRISMA).^15^ The systematic review protocol was registered with the International Prospective Register of Systematic Reviews (PROSPERO; registration number: CRD42020187435). Meta-synthesis was conducted based on the meta-ethnographic approach.^16^ The data management software NVivo 12 for Windows and macOS (Lumivero Operating Systems, Denver, CO; see https://lumivero.com/products/nvivo/) was used to assist in coding.

### Systematic search strategy

A systematic search was first conducted from the inception of this research study until April 2020, and was updated on 6 May 2022, on six online databases: Medline, EMBASE, PsycINFO, CINAHL, Open Dissertations and British Library Ethos. Multiple pilot searches were applied to, and adapted for, each database before running the final systematic searches. These final systematic searches included a combination of ‘Medical Subject Headings’ (MeSH) and text words. Three different clusters of keywords, around ‘self-harm’, ‘South Asian’ and ‘United Kingdom’, were used. The reference lists of the included articles and relevant reviews were also hand-searched to identify further studies. The entire search strategy is reported in Supplementary Appendix 1 available at https://doi.org/10.1192/bjo.2023.63.

### Inclusion and exclusion criteria

Studies were included in the systematic review if they met the following criteria: (a) they employed qualitative or mixed-method designs and presented qualitative findings written in English; (b) they included South Asian people who are UK residents (South Asian countries are Afghanistan, Bangladesh, Bhutan, India, Maldives, Nepal, Pakistan and Sri Lanka; studies with multi-ethnic sample groups were included if they presented findings from South Asian people separately); (c) they examined the views and experiences of South Asian people relating to self-harm, suicidal behaviour or suicidal ideation, regardless of age and gender, although as the current review aims to explore participants’ perceptions of self-harm, personal history of self-harm was not required and participants might have shared their own self-harm history or opinions about how self-harm and suicidal behaviour are experienced in their community; and (d) they reported participants’ views or experiences relating to self-harm or suicide, even if the primary focus of the study was not on self-harm or suicide.

Studies were excluded if they met any of the following criteria: (a) they did not present original and qualitative data; (b) they were conducted outside the UK; (c) they did not include South Asian participants or did not present findings from South Asian participants separately from other ethnicities and (d) they did not specifically discuss self-harm, or suicidal behaviour or ideation.

### Study selection

A two-stage screening process was applied. Initially, two reviewers (B.O.-D., S.K.K.) read the titles and abstracts of the studies and then the full texts independently. At both stages, the reviewers discussed the suitability of the selected studies according to the selection criteria. When there was disagreement over an article, the supervision team (N.H., M.P., S.G.) was consulted. The first reviewer contacted the authors of the papers for further clarification if needed.

### Data extraction

Essential information was extracted from the included studies by the two reviewers (B.O.-D., S.K.K.) independently. Descriptive data from the studies, including the authors, publication year, study type, the study aims, sample size, data collection methods and analysis methods, are presented in Table 1.

**Table 1:** Details of the included studies

Author(s) and year	Type	Title	Aim	Sample (*N*) and gender	Data collection method	Analysis method
Ahmed et al (2007)^27^	Journal article	Self-harm in South Asian women: a literature review informed approach to assessment and formulation	To use available evidence from the literature for guiding assessment and to provide culturally appropriate interventions	1 Female	Interview with open-ended questions	Qualitative, no other information
Aktar (2022)^24^	PhD thesis	The experience of self-harming behaviours that inflict external injuries to the body in UK-based Bangladeshi, Indian, and Pakistani females: An Interpretative Phenomenological Analysis	To explore the experience of self-harming behaviour and the meaning given to this experience	8 Female	Semi-structured interviews	IPA
Bhardwaj (2001)^25^	Journal article	Growing up young, Asian and female in Britain: a report on self-harm and suicide	To explore the reasons why young Asian women self-harmed and to evaluate the service responses they were given	Number not given: all female	In-depth interviews and focus group discussion	Qualitative, no other information
Chantler (2003)^28^	Journal article	South Asian women: exploring systemic service inequalities around attempted suicide and self-harm	To unpack key elements of the complex and layered dynamics at play in working with issues of attempted suicide and self-harm in South Asian women	7 Female	Semi-structured interviews	Qualitative, no other information
Chew-Graham et al (2002)^21^	Journal article	South Asian women, psychological distress and self-harm: lessons for primary care trusts	To encourage Asian women to share their perceptions of experiences of mental distress, attempted suicide and self-harm, and to comment on barriers preventing access to service provision	31 Female in four groups	Focus group discussion	Framework analysis
Gunasinghe et al (2019)^20^	Journal article	Young Muslim Pakistani women's lived experiences of *izzat*, mental health and well-being	To explore how Pakistani women interpret the cultural concept of *izzat*, whether there is interplay between upholding *izzat* and help-seeking	6 Female	Semi-structured interviews	IPA
Hicks and Bhugra (2003)^22^	Journal article	Perceived causes of suicide attempts by UK South Asian women	To describe perceived causes of suicidality by UK South Asian women and to explore marital violence as a possible specific factor in suicidality	43 Female	Focus group discussion	Thematic analysis
Hussain and Cochrane (2003)^29^	Journal article	Living with depression: coping strategies used by South Asian women, living in the UK, suffering from depression	To explore the coping strategies used by Asian women suffering from depression	10 Female	Semi-structured interviews	Grounded theory
Klineberg et al (2013)^23^	Journal article	How do adolescents talk about self-harm: a qualitative study of disclosure in an ethnically diverse urban population in England	To increase understanding about how adolescents in the community speak about self-harm; exploring their attitudes toward and experiences of disclosure and help-seeking	9 Female, 4 male adolescents	Interviews	Thematic analysis using framework approach
Marshall and Yazdani (1999)^32^	Journal article	Locating culture in accounting for self-harm amongst Asian young women	To understand self-harm in relation to ‘professionalised’ approaches to the conceptualisation and understanding of ‘self-harm’ and ‘Asian’ culture, and their interrelationship	7 Female	Semi-structured interviews	Discursive analysis
Mafura and Charura (2022)^33^	Journal article	‘I then had 50 stitches in my arms…such damage to my own body’: An interpretative phenomenological analysis of *Izzat* trauma and self-harm experiences among UK women of South Asian heritage	To explore the lived experience of South Asian women raised in the UK and their perspective of ‘*izzat’*, which refers to honour	12 Female	Semi-structured interviews	IPA
Sambath (2016)^26^	PhD thesis	'The inner scar.' Women's experience of self-harm	To explore how South Asian women from a non-clinical sample experience self-harm and how they describe factors of it	5 Female	Interviews	IPA
Sayal-Bennett (1998)^30^	PhD thesis	Exploring and theorising attempted suicide among Asian women: a qualitative investigation	To explore and privilege participants’ voices, adequately represent their experiences and acknowledge researcher reflexiveness	6 Female	Interviews	Grounded theory
Thabusom (2005)^34^	PhD thesis	Mental health and Asian women: a qualitative study of women's experiences	To explore Asian women's experience of self-harm and attempted suicide	1 Female	Interviews	IPA
Wood (2001)^31^	PhD thesis	Exploring experiences and meanings of self-harm in South Asian women in the UK	To explore the experiences and meanings of South Asian women who self-harm; and their experiences and perceptions of support services	6 Female	Semi-structured interviews	IPA

IPA, interpretative phenomenological analysis.

### Risk-of-bias assessment

Two reviewers (B.O.-D., S.K.K.) assessed the quality of the selected studies independently. The Critical Appraisal Skills Programme (CASP) Qualitative Checklist was used for this assessment.^17^

### Meta-ethnographic synthesis

Meta-ethnography is an interpretative approach for synthesising qualitative studies that was first proposed by Noblit and Hare.^16^ Unlike the traditional aggregative method that summarises the findings of original studies, meta-ethnography helps to produce new understandings and reconceptualisations of phenomena.^16^

Meta-ethnography includes three different syntheses: (a) reciprocal translations of analogous studies, i.e. comparing the similarities between studies; (b) reputational translations of contradictions between studies and (c) a line of argument that interprets any similarities and dissimilarities as new inferences.^16^ In this review, the included studies have sufficient commonalities, rather than disagreements, to enable the application of reciprocal translations.

Reciprocal translations are applied and presented via the notion of first-, second- and third-order constructs.^18^ The first-order constructs are the participants’ quotes in the included studies, the second-order constructs are the authors’ interpretations of these quotes and the third-order constructs are the overarching themes produced from the previous constructs by the research team.^19^ The themes and the constructions produced are presented in Tables 2, 3, and 4.

**Table 2:** The reciprocal translations for ‘Behind self-harm’

Third-order construct	Second-order construct	First-order construct (examples from quotes)
Negative perceptions of self	Low self-esteem and low self-worth^24,25,27–31^ Self–hate^21,24–26^	‘I don't know why I can't love myself, because … maybe I can see myself as a disappointment, like the family see me as a disappointment.’^30^ ‘I just remembered one of the main reasons I used to self-harm it was just self-esteem problem.’^24^
Isolation	Lack of English proficiency^21,22,27,29–31^ Loss of relationship^22,26,30,32–34^	‘Just not being able to speak to them, or understand, that makes me upset.’^21^ ‘I was in a world of my own suffering the hurt in silence.’^34^
Exposure to violence	Racism^21,24,26,30,31^ Abuse and domestic violence^21,22,24,25,28,30–33^	‘We're different, we're treated differently by our own because we're women, we're treated differently outside because we're Asian.’^21^ ‘I was bullied in school, in primary school. I remember vague bits of it of having kids say racist things to me … I remember making up like rhymes about killing my family and stuff.’^24^
Economic and political inequalities	No welfare rights^28^ Poverty^27,30^ Housing issue^28,30^	‘I haven't got stay [right to remain in this country] I can't get benefits. I didn't ask for any money from my husband. There should be some kind of support. What about those women who don't have family here?’^28^ ‘I mean, when house is gone and the court people say you have to empty the house on that day and I bring up such a good family and I have to go to refuge house to live with my children, how do I feel that time?’^30^
Cultural risk factors	Gender inequality^21,24,26,33^ Unrealistic expectations^21,24–26,31–34^ Community grapevine^21,33^ Family honour^20,21,25,31–34^ Being controlled^20,21,24–26,28,30–34^	‘I don't really want to go back to Pakistan, to listen to all the people talking about me. That kills you more, even though you are alive, you're almost dead, inside you are death. People blame the women – not the man, the woman is guilty.’^28^ ‘I mean, protecting the honour of the family is another expectation.’^26^ ‘My mum, she was controlling, like ‘Oh, you can't go uni’ then I self-harmed.’^31^

**Table 3:** The reciprocal translations for ‘Functions of self-harm’

Third-order construct	Second-order construct	First-order construct (examples from quotes)
Emotion regulation	Relief from pain^24–26,30–34^ Releasing the anxiety^29^	‘At that one moment I just get bad, I get really bad that I want to just ease the pain.’^30^ ‘I think being quite young I just didn't have … I didn't have the vocabulary to express myself. Um I didn't have anyone to express myself to so it was just, again, a way of expressing myself.’^26^
Communication and expression	Expression of anger, distress, and emotional pain^20,21,23,24,26,31–33^ Compensation of lack of words for communication^26^	‘I was angry. It's always been because I was like angry … when you get really angry and you don't know what to do and that's the only thing (self-harm) there is to do. So that's why I used to do it.’^31^
Control	Taking control^25,26,31–33^ Losing control over self-harm^24,31,32^	‘While I was cutting I felt more in control, whereas before I'd cut I sort of felt like “Oh God”, there's nothing I can control.’^32^ ‘ … So, I start hurting myself and it just got to a place where I couldn't stop.’^24^
Provoking change	The purpose of being seen^23^ Desire to care^23,31^ Trigger external support^25,26^	'But at least doing that [self-harm], I’ve got some counselling ... at least I’ve got somewhere, I can come and talk, unload a little bit. But unless ... if I hadn ’t done that ... I don’t think I would even have had that and I would have lost all channels of contact with everyone outside.'^25^ ‘They're always going to be there for me but that's only been enforced after I've acted out.’^26^
Negative experience	Feeling shame^24,26,31,33^ Self-punishment^26,33^ Unhelpful^26,30,32^ Sinful^24,31^	‘ … I would cut, like I said, it was like a punishment for me.’^25^ ‘Awful, coz it's against our religion. We're not supposed to harm our body. Because we see it as a gift.’^31^ ‘Then I thought why I've done this like it's not worth it like why am I doing this to myself.’^24^

**Table 4:** The reciprocal translations for ‘Recovery from self-harm’

Third-order construct	Second-order construct	First-order construct (examples from quotes)
Self-help	Volunteering^30^ Religion^20,27,29,30,34^	‘ … Two things that have made it easy for me, is number one a very supportive family. Number two, knowing that Islam won't allow me to oppress myself, nor others … ’^20^
Positive service experience	Having good counselling^30,31,34^ Community groups^28^	‘I had a good treatment and I got no complaints, it's like a family to me.’^30^
Negative service experience	Barriers to access: Confidentiality concern^21,25,26,31^ Unaware of available services^30^ Inadequate service response: Lack of cultural understanding^21,25,26,31,33^	‘Even after my suicide [attempt] . . . I experienced quite a few professionals whose approach to it all is just go back to your family. I even had one who told me to get married.’^25^ ‘When I ‘cut’ before, when I was admitted to [xxx Hospital] and I explained to the staff why I had done it and that it was all to do with *Izzat* …. they wrote in my notes that I was losing touch with reality. I wished I had someone who would understand my needs and what *Izzat* is about.’^33^
Suggestions for improvement of mental health services	Raising awareness^21,25,26,31^ Building trust to helping agencies^21^ Advertising available health services^21,25,26,32^ Helplines^23,28,31,32^ Addressing confidentiality concern^23,32^ Working with educational context^26^	‘But it's hard, like … my mum watching or my brother watching me, or someone like that. So it's kind of hard to say, call up and speak to someone in front of somebody else, when it's supposed to be confidential … So, I think if they are on-line, probably just emailing or talking to someone online … that's better.’^23^ ‘Skills, job so they can earn money. Something like a day centre so they can build their future up.’^30^

## Result

**Fig. 1:**
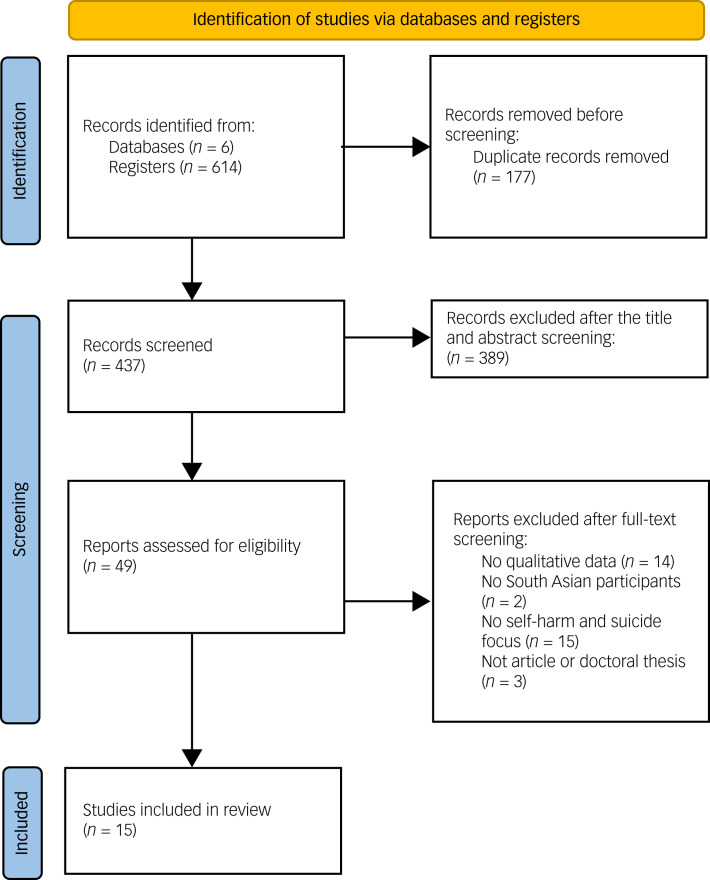
Preferred Reporting Items for Systematic Reviews and Meta-Analysis (PRISMA) flow diagram.

A total of 614 articles were identified by the searches. After removing duplicates, 437 studies remained. Two reviewers independently screened the titles and abstracts of these studies, of which 49 studies underwent full-text screening. Finally, ten articles and five doctoral theses were included in the meta-synthesis. The literature search flow diagram is presented in Fig. 1.

### Descriptive characteristics of the selected studies

Table 1 presents the key descriptive characteristics of the included studies. Twelve studies recruited participants with lived experience, whereas three studies presented the experiences and perceptions of self-harm and suicide of people with and without lived experience.^20–22^ Of the 15 articles, 14 had female participants, and only one study^23^ included both female and male participants. Although mean age was not recorded in all of the included studies, the research population in most studies included young adults, with the overall age range being 14–55 years.

### Risk-of-bias assessment

The results of applying the CASP Qualitative Checklist showed that most studies had clear aims and rigorous methods for data collection and analysis. However, five studies did not discuss ethical considerations and seven studies did not report the researchers’ reflections. In all of the doctoral theses, ethical approval was obtained and the data collection process and results were clearly presented. Results of the entire quality assessment are available in Supplementary Appendix 2.

### Main findings of meta-synthesis

The meta-synthesis of the included studies was generated based on three reciprocal translations, which are behind self-harm, functions of self-harm and recovery from self-harm. The reciprocal translation of ‘behind self-harm’ explores the associations between risk and precipitating factors, and self-harm. ‘Functions of self-harm’ represents the descriptions and meanings of self-harm for the participants. ‘Recovery from self-harm’ discovers self-help, professional help received and suggestions for the improvement of mental health services.

#### Behind self-harm

Behind self-harm uncovers what might have led people to harm themselves. Several factors were directly linked to self-harm, in addition to the factors indirectly leading to self-harm by causing chronic stress and difficulties in an individual's life. These direct and indirect risk factors spanned personal, interpersonal and societal matters. Table 2 shows the reciprocal translations of ‘behind self-harm’, which include five third-order constructs: negative perceptions of self, isolation, exposure to violence, cultural risk factors and economic problems.

Negative perceptions of self were discussed in seven studies. Self-harm was directly linked to self-hate,^21,24–26^ self-worth and low self-esteem.^24,25,27–31^ Those who reported self-hate saw self-harm as self-punishment.^26^

Isolation and its impact on mental health were mentioned in almost all studies. Some studies explored how conceptual factors led to increased isolation in South Asian women.^21^ The lack of trusted companions was a commonly reported theme. Therefore, South Asian women were unable to share their difficulties, which led to ongoing distress. Also, interpersonal losses and rejections were linked to suicidal ideation and behaviours.^22,26,30,32^ The lack of English proficiency was another factor that was associated with a lack of knowledge of health services. This prevented individuals from accessing mental health services and from knowing about available resources and their rights.^21,22,27,29–31^

Exposure to violence represented all kinds of experiences of racist, sexist and abusive behaviours exacerbating mental well-being, which were reported in the studies. Racism was reported as an external pressure by South Asian women.^21,24,26,28,30,31^ Abusive relationships and domestic violence were linked in most studies to self-harm or suicide attempts when women could not receive any assistance to deal with their domestic situations.^21,22,24–26,28,30–33^ Discrimination owing to gender and ethnicity also increased isolation among South Asian women.^21^ South Asian women have spoken about this double jeopardy they have faced:

‘We're different; we're treated differently by our own because we're women, we're treated differently outside because we're Asian’.^21^

Economic and political inequalities were reported in relation to housing issues, poverty and limited access to benefits/welfare rights.^27,28,30^ These issues were discussed in relation to systemic and political aspects such as the 2-year rule. The 2-year rule was a former law that mandated the deportation of foreign spouses who ended their marriage within 2 years of coming to the UK through marriage. This policy had negative outcomes of leaving some immigrant women in abusive marriages with limited options to leave or receive financial help.^28^ For example, South Asian women who do not have welfare rights become economically dependent on their relatives, which perpetuates their distress when they experience domestic violence.^28^ ‘State and familial oppression’ causes such distress, and has resulted in self-harm and suicide.^28^ In one woman's account, the distress caused by the issue of obtaining a visa was presented as follows:

‘I think about it [suicide] a lot; the last two weeks have been awful – will I get my visa or not?’.^28^

Further, the failure to provide housing to women who have escaped abusive relationships was also reported as a risk factor for suicide. Women could be easily tracked by their abusers in small communities, which makes providing safe housing to domestic survivors more crucial.^28^

Cultural risk factors were discussed in most of the studies, including gender inequality, unrealistic expectations set for women, the community grapevine, family honour, forced marriage and being controlled by family members. There are some cultural risk factors for mental health that are not specific to South Asian culture, such as gender inequality. However, personal and social resources for dealing with such external pressures might differ among ethnic minority women.

Gender inequality is reported as feeling oppressed by ‘gender-based roles and expectations’,^26^ and discrimination toward women might cause distress and self-harm.^21,24,33^ South Asian women are subjected to the community grapevine as their community tends to monitor and blame women.^21^ Unrealistic expectations were reported as another external pressure on women; these included academic expectations in families and traditional South Asian expectations of family members and the community.^21,24–26,31–34^ Traditional expectations set high standards for women to follow. For example, women should protect their family's prestige with their academic achievements and ‘correct’ behaviours.^20,21,24–26,31–34^ This interacts with another theme, family honour. Family honour is called *izzat* in South Asian culture, and is defined as ‘family or personal honour/respect, or as status and prestige in the eyes of the community’.^21^ Giving much importance to family honour might cause people to ignore women's struggles and to hide the abuse within the family, all of which leave women alone with their distress.^20,33^ Being controlled was another cultural risk factor for distress mentioned in many studies.^20,21,24–26,28,30–34^ Forced marriage, being prevented from having an education and lack of autonomy were some examples of controlling behaviours. All unequal behaviours toward women listed above set barriers to sharing difficulties with others and seeking help from outside of the community.^21^ Consequently, women had limited space and resources to discuss their difficulties and solve their problems, which caused further isolation, depression and self-harm.^20^

#### Functions of self-harm

In the literature, functions of self-harm and reasons for self-harm were used interchangeably.^35^ In the current review, we define ‘functions of self-harm’ as how self-harm was perceived and what meanings and motivations were attributed to it. The theme of functions of self-harm was elaborated from the participants’ comments of what had happened just before, during or after self-harm. Table 3 shows the five third-order constructs that were generated from the findings of the studies: emotion regulation, communication and expression, taking or losing control, provoking change and negative experience.

Emotion regulation refers to the temporary positive effects of self-harm in easing the burden of a situation. In most studies, this situation was synthesised as a coping mechanism. Specifically, self-harm was viewed as relief from emotional pain.^24–26,30–34^ Self-harm functions to convert emotional pain into physical pain and, consequently, to produce a sense of relief just afterward.^31,32^ Among women with depression, self-harm was seen as a way of releasing anxiety.^29^

Communication and expression associated with self-harm was seen as the expression of anger, emotional pain and distress.^20,21,23,24,26,31–33^ Self-harm was also described as a way of communicating when there is a lack of words to describe emotions.^26^ Also, the intention to be noticed by others was reported.^23,31^ This is beyond attention-seeking and is a sign of unspoken struggles.^31^

‘The thoughts I used to have … after cutting myself and before cutting myself, were, like, just show somebody … I wasn't a good talker, like, back then, so … that's why I knew that they would kind of help me in some way’.^23^

Control was mentioned when describing self-harm in different ways. Self-harm was seen as taking control when participants felt that they had no control or power over their lives.^25,26,31–34^ However, self-harm might mean losing control when it becomes a daily practice or only an option for temporary positive side-effects, such as feeling relief. Some participants reported that they could not stop self-harming even if they wanted to, and that they lost control of the frequency of self-harm.^24,31,32^

Provoking change represents the theme of self-harm being used to change situations or others’ behaviour. For some, self-harm was a way of obtaining care from others,^23,31^ including external professional support.^25,26^

In terms of negative experience, self-harm was described as having negative features and consequences. Self-harm was attached to feelings of shame and guilt afterward,^24,26,31,33^ rather than bringing real change.^30,32^ It was also described as unhelpful and sinful.^24,26^

#### Recovery from self-harm

Recovery from self-harm included self-help, positive and negative mental health service experiences, and suggestions for improving mental health services, which are presented in Table 4.

Self-help represents the second-order constructs of religion and volunteering. Religion was mentioned as a positive aspect of managing unbearable circumstances. Believing in Islam, which does not permit any harm to the self, praying to Allah and accepting the faith helped the participants to deal with their problems.^20,27,29,30,34^ Doing voluntary work provided the participants with some social gain, such as helping others, spending their time well and getting away from difficult home situations.^30^

Positive health service experiences included receiving good counselling and community services support. The features of good counselling were described as ‘being listened to and understood’ with empathy by professionals who encourage women to express themselves.^34^ Cognitive–behaviour therapy was helpful because it provided ‘practical solutions’.^31^ Some participants explicitly stated that having counselling prevented them from attempting suicide^30^ and self-harm.^31^ Community services were valued because they provided mental health support, offered housing and benefits support, and made effective referrals to the appropriate agencies.^28^

Negative health service experiences were presented in terms of barriers to accessing services and inadequate service responses. Mistrust of health services was one of the barriers to seeking help. Participants reported that they would mistrust professionals from outside their community as these professionals might not understand them because of limited knowledge of South Asian culture.^21,25,26,31^ Similarly, they mistrusted professionals from within their community, who might break confidentiality or judge them according to traditional views.^21,25,26,31^ Further, lack of knowledge about available services was another barrier to obtaining help.^25,28^ Some studies evaluated service responses to self-harm and suicide. Primary care workers provided limited support because of their lack of understanding of self-harm^32^ and non-referral of patients to mental health services.^28^ Some respondents had experienced health workers not listening with empathy and understanding.^28^

Suggestions for improving access to support services and service responses were synthesised. To improve mental health access, South Asian women recommended that trust in healthcare services and professionals should be built to enable South Asian women to discuss their personal concerns.^21^ Also, the stigma around help-seeking and self-harm should be reduced.^26,31,32^ This could be achieved by increasing awareness about psychology and mental health,^21,25,26,31^ along with promoting knowledge of self-harm and of the services available in the community and in educational institutions.^21,25,26,32^ For instance, support groups in schools could create a space to talk about self-harm and mental health openly.^26^ The availability of services should be increased by providing telephone or online helplines.^23,28,31,32^

For the improvement of service responses, the participants suggested that health professionals should have a greater understanding of the socioeconomic and cultural aspects of mental health among ethnic minority communities.^26,33^ Moreover, mental health services should address privacy and confidentiality concerns among patients.^23,32^ Ethnic minority patients’ preferences about health professionals’ ethnicity and gender should also be taken into account.^32^

## Discussion

This is the first systematic review and meta-synthesis to explore South Asian people's experience and understanding of self-harm and suicidal behaviour in the UK. There are three main findings from our analysis. First, socioeconomic, political and cultural factors are found to be major stressors for South Asian women who have faced a double jeopardy of discrimination owing to their gender and ethnicity, from both within and outside their communities. Second, our findings on functions of self-harm are in line with the most studied function models in the self-harm literature, such as affect regulation and self-punishment. There are remarkable similarities regarding functions of self-harm by South Asian people with the wider literature on White majority self-harm samples. Therefore, it is important not to place too much emphasis on stereotypical assumptions about the cultural components of self-harm in South Asian people. Third, recovery from self-harm involves self-care activities, protective factors and professional help. Suggestions for improvement in engagement with mental health services are also generated.

Our analysis has identified several personal and interpersonal reasons and risk factors for self-harm, such as negative perceptions of self and relationship problems. This theme is consistent with recent research findings, which have shown lower self-esteem and interpersonal conflict to be linked to self-harm.^36,37^

The impact of socioeconomic and cultural factors on self-harm among South Asian people should be seriously taken into the account, which also aligns with the previous research.^38,39^ South Asian women have been subjected to multilevel stressors by the misuse of South Asian cultural values, such as setting high social expectations for women and controlling women through family honour.^40^ Further, racism has been associated with common mental health difficulties in ethnic minority groups in the UK.^38,39,41^ Our findings also reveal that financial problems have a considerable influence on the mental health of South Asian people in the UK.^42^ Although the impact of economic difficulty on mental health has been acknowledged nationally, ethnic minorities seem to be more disadvantaged because of uncertain residency status or language barriers.^12,42^ Longstanding economic and health inequalities also appeared during the COVID-19 pandemic.^43^ The rates of abuse, self-harm and suicidal thoughts were greater in women and Black, Asian and minority ethnic groups, along with some other socioeconomically disadvantaged groups.^44^

Further, limited immigration policies to protect South Asian women against domestic violence cause further isolation and mental health difficulties.^45,46^ A recent systematic review of South Asian women who have experienced domestic violence in high-income countries states that multilayer barriers to seeking help include insecure immigration status, limited governmental and third-sector support, and lack of financial support.^47^

Our analysis has captured functions of self-harm that are aligned with previous systematic reviews.^48,49^ Empirical literature on the functions of self-harm were reviewed by Klonsky,^49^ and seven common function models were proposed. Our analysis captures four of these seven function models: affect regulation, self-punishment, interpersonal influence and interpersonal boundaries.^49^ Affect regulation is the most common function of self-harm in the literature, and means that self-harm works to manage negative emotions.^48,49^ Moreover, self-harm as a means of communication or expression was a notable function in our findings.^48^ This may be explained by our findings about increased isolation owing to socioeconomic and cultural contexts that are discussed under reasons for self-harm. Limited personal or professional resources in terms of talking about emotional struggles could be associated with self-harm being a way of expressing emotions.

The self-harm recovery process includes multiple personal and social qualities such as volunteering, believing in a religion, support from family and friends and having access to good counselling and community services, all of which are in line with a recent review on cessation of self-harm.^50^ On the other hand, barriers to accessing and benefitting from professional help include a lack of awareness of available services, the stigma against seeking psychological help, confidentiality concerns and a lack of trust in health professionals.^51,52^

### Strengths and limitations

This is the first meta-synthesis using on the meta-ethnography approach to explore the views and experiences of self-harm among South Asian people in the UK. We have included both grey and published literature in our review. Another strength of this study is that an explicit definition of the ethnic group being focused on was applied as an inclusion criterion. This allowed us to acquire an in-depth understanding of the experiences of self-harm among South Asian people. On the other hand, considering all South Asian people as one ethnic group could cause nuanced variations among South Asian communities to be missed. Therefore, we acknowledge the uniqueness of mental health experiences among South Asian people according to their personal differences, nationalities, migration stories and faith. Further, because of the subjective nature of qualitative synthesis, the researchers’ interpretations of the data could be influenced by their own cultural and professional backgrounds. To challenge this, we provided references from the selected studies in relation to each theme, which increases the credibility of the meta-synthesis.^53^

One of the limitations of the included studies is that very little attention has been paid to the perceptions of South Asian men about self-harm and suicide. Only one study included male participants. Additionally, the included studies are limited to the young adult research population, so there is also a need to explore self-harm among older South Asian adults. Finally, only five of the 15 studies were conducted during the past decade. Therefore, up-to-date research should be undertaken to explore self-harm among South Asian people, including all gender groups and older adults.

### Clinical and research implications

This study provides important implications for researchers, mental health professionals and commissioners in improving treatments for, and the prevention of, self-harm and suicide in South Asian communities in the UK. We offer four critical recommendations: (a) training for care providers in culturally sensitive practice, (b) implementing of culturally adapted psychosocial interventions, (c) raising of mental health awareness among South Asian communities and (d) increasing access to mental health services.

Cultural understanding and sensitivity among mental health workers should be improved through training and supervision specific to working with ethnic minority groups.^54,55^ Our research highlights socioeconomic and cultural aspects of self-harm among South Asian people. These findings can inform care provider training on culturally sensitive practice when working with South Asian people. Moreover, culturally adapted psychological interventions were found to be effective treatments in recent meta-analyses.^56,57^ Our findings strongly encourage the development and evaluation of culturally sensitive self-harm interventions for South Asian people by using evidence that identifies the unmet needs of South Asian people in mental health services.

Further, mental health awareness should be raised among South Asian people through community-based programmes and conversations on confidentiality concerns. This is especially needed in relation to seeking help for self-harm, as shame and secrecy are commonly associated with experiences of self-harm and suicidal behaviours.^58,59^ In addition, community organisations can be enhanced to provide long-term and practical support, such as life coaching, safety from abusive environments and financial assistance.

Increasing accessibility to mental health services might involve promoting these services, extending the availability of helplines and offering flexible timings and childcare to parents or caregivers. Online interventions or recorded psychoeducation would be helpful to those who cannot physically access health services.^60,61^

## Data Availability

Data availability is not applicable to this article as no new data were created or analysed in this study.

## Supplementary material

For supplementary material accompanying this paper visit http://doi.org/10.1192/bjo.2023.63. click here to view supplementary material
