# Supplementary material for: Understanding self-harm and suicidal behaviours in South Asian communities in the UK: systematic review and meta-synthesis
Source: BJPsych Open. 2023 May 15;9(3):e82. doi: 10.1192/bjo.2023.63 (PMC10228242; doi:10.1192/bjo.2023.63)
Supplement: Supplementary file 1 [file bjosup.zip › S2056472423000637sup001.docx]

| Appendix: 1 Systematic Search Strategy | | |
| --- | --- | --- |
| Database: Embase <1980 to 2022 Week 17> APA PsycInfo <1806 to May Week 1 2022> Ovid MEDLINE(R) and Epub Ahead of Print, In-Process, In-Data-Review & Other Non-Indexed Citations, Daily and Versions <1946 to May 05, 2022> | | |
| # | Query | Results from 6 May 2022 |
| 1 | self-harm*.mp. | 25,392 |
| 2 | self-injur*.mp. | 30,631 |
| 3 | self-poison*.mp. | 5,709 |
| 4 | self-cut*.mp. | 781 |
| 5 | self-burn*.mp. | 169 |
| 6 | self-mutil*.mp. | 8,615 |
| 7 | self-destruct*.mp. | 10,427 |
| 8 | DSH.mp. | 3,083 |
| 9 | NSSI.mp. | 4,847 |
| 10 | suicid*.mp. | 326,529 |
| 11 | 1 or 2 or 3 or 4 or 5 or 6 or 7 or 8 or 9 or 10 | 363,941 |
| 12 | South Asia*.mp. | 33,730 |
| 13 | Bangl*.mp. | 48,272 |
| 14 | Pakista*.mp. | 81,883 |
| 15 | India*.mp. | 630,061 |
| 16 | Bhutan*.mp. | 3,008 |
| 17 | Sri Lanka*.mp. | 24,174 |
| 18 | Afghanistan*.mp. | 20,605 |
| 19 | Maldives*.mp. | 1,107 |
| 20 | Nepal*.mp. | 34,571 |
| 21 | 12 or 13 or 14 or 15 or 16 or 17 or 18 or 19 or 20 | 829,139 |
| 22 | United Kingdom.mp. | 797,356 |
| 23 | UK.mp. | 430,726 |
| 24 | England/ or England.mp. | 269,252 |
| 25 | Brit*.mp. | 287,108 |
| 26 | Wales.mp. | 83,522 |
| 27 | Welsh.mp. | 6,919 |
| 28 | Ireland.mp. | 233,393 |
| 29 | Irish.mp. | 31,724 |
| 30 | Scot*.mp. | 137,836 |
| 31 | 22 or 23 or 24 or 25 or 26 or 27 or 28 or 29 or 30 | 1,815,732 |
| 32 | 11 and 21 and 31 | 454 |
| 33 | 11 and 21 and 31 | 454 |
| 34 | self-harm*.mp. | 25,392 |
| 35 | self-injur*.mp. | 30,631 |
| 36 | self-poison*.mp. | 5,709 |
| 37 | self-cut*.mp. | 781 |
| 38 | self-burn*.mp. | 169 |
| 39 | self-mutil*.mp. | 8,615 |
| 40 | self-destruct*.mp. | 10,427 |
| 41 | DSH.mp. | 3,083 |
| 42 | NSSI.mp. | 4,847 |
| 43 | suicid*.mp. | 326,529 |
| 44 | 34 or 35 or 36 or 37 or 38 or 39 or 40 or 41 or 42 or 43 | 363,941 |
| 45 | South Asia*.mp. | 33,730 |
| 46 | Bangl*.mp. | 48,272 |
| 47 | Pakista*.mp. | 81,883 |
| 48 | India*.mp. | 630,061 |
| 49 | Bhutan*.mp. | 3,008 |
| 50 | Sri Lanka*.mp. | 24,174 |
| 51 | Afghanistan*.mp. | 20,605 |
| 52 | Maldives*.mp. | 1,107 |
| 53 | Nepal*.mp. | 34,571 |
| 54 | 45 or 46 or 47 or 48 or 49 or 50 or 51 or 52 or 53 | 829,139 |
| 55 | United Kingdom.mp. | 797,356 |
| 56 | UK.mp. | 430,726 |
| 57 | England/ or England.mp. | 269,252 |
| 58 | Brit*.mp. | 287,108 |
| 59 | Wales.mp. | 83,522 |
| 60 | Welsh.mp. | 6,919 |
| 61 | Ireland.mp. | 233,393 |
| 62 | Irish.mp. | 31,724 |
| 63 | Scot*.mp. | 137,836 |
| 64 | 55 or 56 or 57 or 58 or 59 or 60 or 61 or 62 or 63 | 1,815,732 |
| 65 | 44 and 54 and 64 | 454 |
