# Supplementary material for: Understanding self-harm and suicidal behaviours in South Asian communities in the UK: systematic review and meta-synthesis
Source: BJPsych Open. 2023 May 15;9(3):e82. doi: 10.1192/bjo.2023.63 (PMC10228242; doi:10.1192/bjo.2023.63)
Supplement: Supplementary file 1 [file bjosup.zip › S2056472423000637sup002.docx]

Appendix 2: Risk of Bias Assessment

CASP Questions and Scores

| Appendix 2: Risk of Bias Assessment | | | | | | | | | | | | |
| --- | --- | --- | --- | --- | --- | --- | --- | --- | --- | --- | --- | --- |
|  | Scores | | | CASP Questions and Scores | | | | | | | | |
| Authors | Total Yes | Total Can’t Tell | Total No | 1. Was there a clear statement of the aims of the research? | 2. Is a qualitative methodology appropriate? | 3. Was the research design appropriate to address the aims of the research? | 4. Was the recruitment strategy appropriate to the aims of the research? | 5. Was the data collected in a way that addressed the research issue? | 6. Has the relationship between researcher and participants been adequately considered? | 7. Have ethical issues been taken into consideration? | 8. Was the data analysis sufficiently rigorous? | 9. Is there a clear statement of findings? |
| Ahmed et al., 2017 | 6 | 4 | 0 | Y | Y | Y | C | Y | C | C | C | Y |
| Aktar, 2022 | 10 | 0 | 0 | Y | Y | Y | Y | Y | Y | Y | Y | Y |
| Bhardwaj et al., 2001 | 8 | 2 | 0 | Y | Y | Y | Y | Y | C | C | Y | Y |
| Chantler et al., 2003 | 8 | 2 | 0 | Y | Y | Y | Y | Y | C | C | Y | Y |
| Chew-Graham et al., 2002 | 10 | 0 | 0 | Y | Y | Y | Y | Y | Y | Y | Y | Y |
| Gunasinghe et al., 2019 | 9 | 1 | 0 | Y | Y | Y | Y | Y | C | Y | Y | Y |
| Hicks & Bhugra 2003 | 6 | 4 | 0 | C | C | Y | Y | Y | C | C | Y | Y |
| Hussain & Cochrane 2003 | 9 | 1 | 0 | Y | Y | Y | Y | Y | Y | C | Y | Y |
| Klineberg et al., 2013 | 9 | 1 | 0 | Y | Y | Y | Y | Y | C | Y | Y | Y |
| Marshall & Yazdini, 1999  Mafura & Calvin, 2021 | 9  9 | 1  1 | 0  Y | Y  Y | Y  Y | Y  Y | Y  Y | Y  Y | C  C | Y  Y | Y  Y | Y  Y |
| Sambath, 2016 | 10 | 0 | 0 | Y | Y | Y | Y | Y | Y | Y | Y | Y |
| Sayal Bennet, 1998 | 10 | 0 | 0 | Y | Y | Y | Y | Y | Y | Y | Y | Y |
| Thobusom, 2005 | 10 | 0 | 0 | Y | Y | Y | Y | Y | Y | Y | Y | Y |
| Wood, 2001 | 10 | 0 | 0 | Y | Y | Y | Y | Y | Y | Y | Y | Y |
